# Supplementary material for: Ultrasound system for precise neuromodulation of human deep brain circuits
Source: Nat Commun. 2025 Sep 5;16:8024. doi: 10.1038/s41467-025-63020-1 (PMC12413462; doi:10.1038/s41467-025-63020-1)
Supplement: Supplementary file 1 — Supplementary Information [file 41467_2025_63020_MOESM1_ESM.pdf]

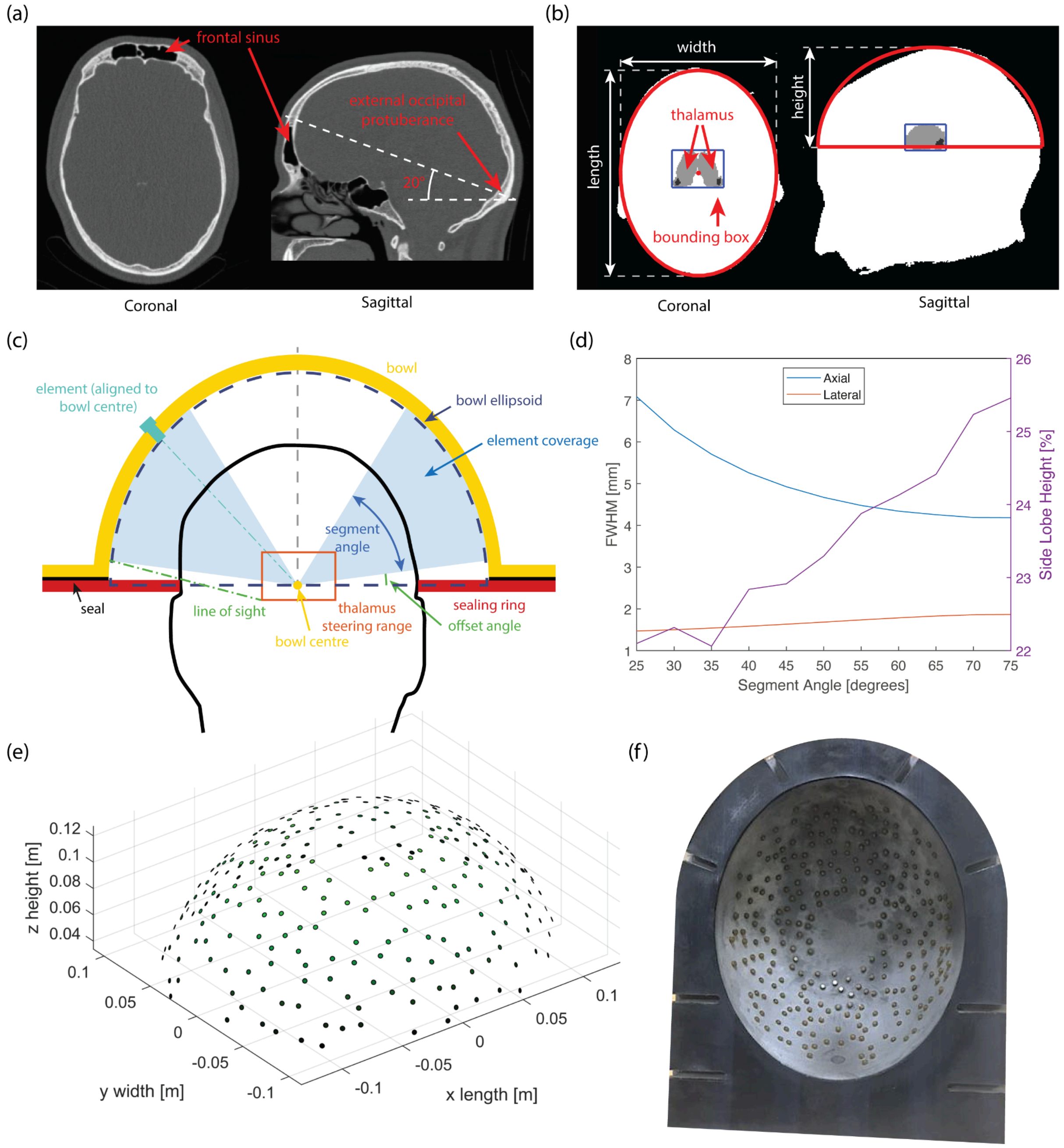

Figure S1: Design and dimensioning of the transcranial ultrasound helmet
The helmet size was based on analysis of images from 16 healthy participants taking into account their head orientation in the scanner. **(a)** Illustrative CT image showing the chosen inclination angle of 20° and the approximate positions of the frontal sinus and external occipital protuberance for one participant. **(b)** Head size was determined for each participant by fitting an ellipse. The average ellipse dimensions were 206 × 157 × 96 mm (length × width × height). **(c)** The elements were selected to only cover a subset of the helmet ensuring line of sight to the average thalamus bounding box. **(d)** The segment angle was chosen to balance focal size with side lobe height. **(e)-(f)** Final element positions. The elements are randomly distributed on an ellipsoidal bowl with dimensions 286 × 237 × 135 mm (40 mm offset from average head ellipsoid), with a segment angle of 55°, and an offset angle of 15°. Two exclusion zones are also used to allow water connections at the highest and lowest points on the bowl.

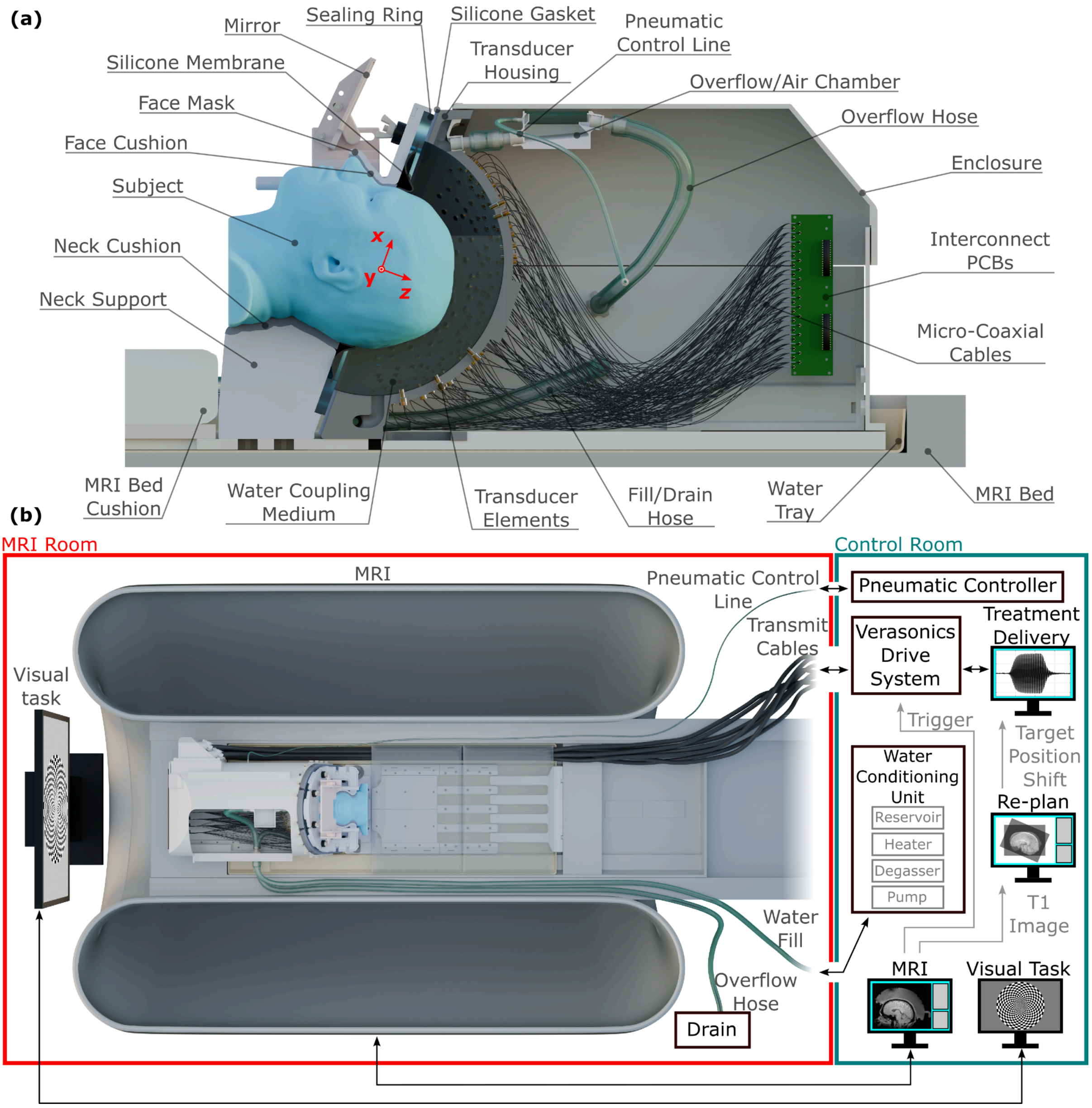

Figure S2: Technical implementation of the MR-compatible ultrasound system
(a) Cross sectional view of the advanced transcranial ultrasound system. The participant is coupled to the transducer elements via a water layer, which is retained using a silicone membrane around the participant's head. A gasket around the transducer housing flange prevents leakage. The transmit signals are routed via interconnect PCBs to the individual transducer elements. **(b)** Schematic view of the transcranial ultrasound system installed in the MRI, allowing concurrent neuromodulation and functional neuroimaging. Supporting equipment is located in the MRI control room. The water conditioning unit supplies water to the system via hoses. The transducer elements are connected to the Verasonics via 8 m cables. A pneumatic controller is used to restrict the hydrostatic pressure on the participant's head, via an airline.

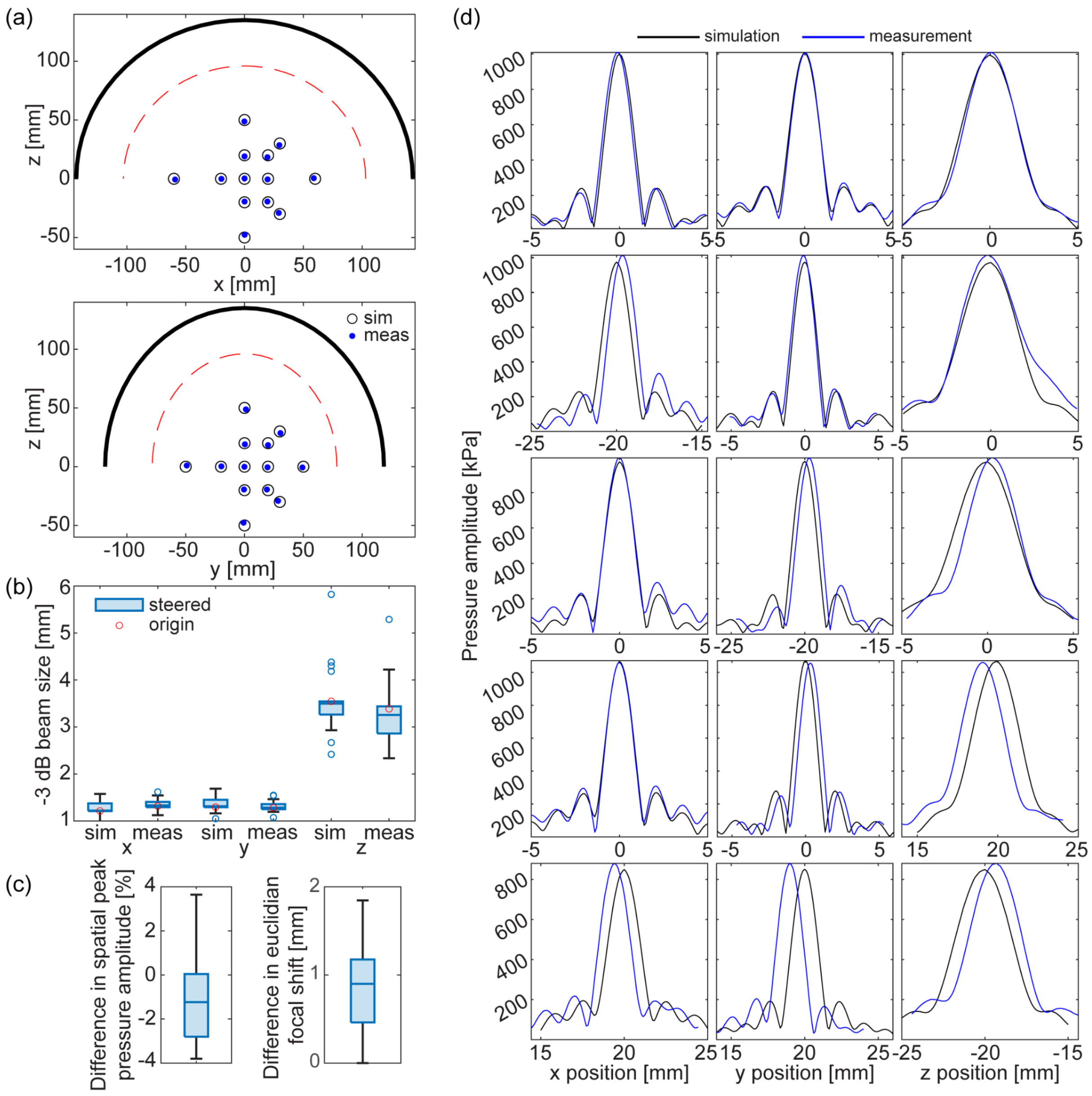

Figure S3: Free field validation of ultrasound focal steering capabilities
(a) Locations of steered free field validation measurements in the xz (top) and yz (bottom) planes. The bowl surface is shown in black and the average head by the red dashed line. Simulated focal positions using phases calculated geometrically are shown by black circles, blue dots show the position of the corresponding measured field steered using the same geometric phases. Measurement locations span the brain for the average head size. **(b)** Median and range (25th - 75th percentile) of simulated and measured free field -3 dB beam dimensions across 13 steering positions. Red circles show the focal dimensions at the origin, blue circles show outliers. Focal dimensions are preserved across the steering range. **(c)** Median difference between measured and simulated spatial peak pressure amplitude was -1.23%, and the difference was less than 4% across all positions. This demonstrates the accuracy of the transducer definition used in simulation, and the close to ideal performance of the array. Difference in focal shift is calculated from the absolute difference between the intended shift and the euclidean distance from the origin to the measured focal position. The median difference was 0.9 mm, and the difference was less than 2 mm across all positions. **(d)** Simulated (black) and measured (blue) pressure amplitude profiles through the focus in water at the origin, (0, 0, 0) mm, and for steering locations (-20, 0, 0) mm, (0, -20, 0) mm, (0, 0, 20) mm and (20, 20, -20) mm. A drive voltage of 8 V was used for all measurements, resulting in a spatial peak pressure of 1 MPa at the origin.

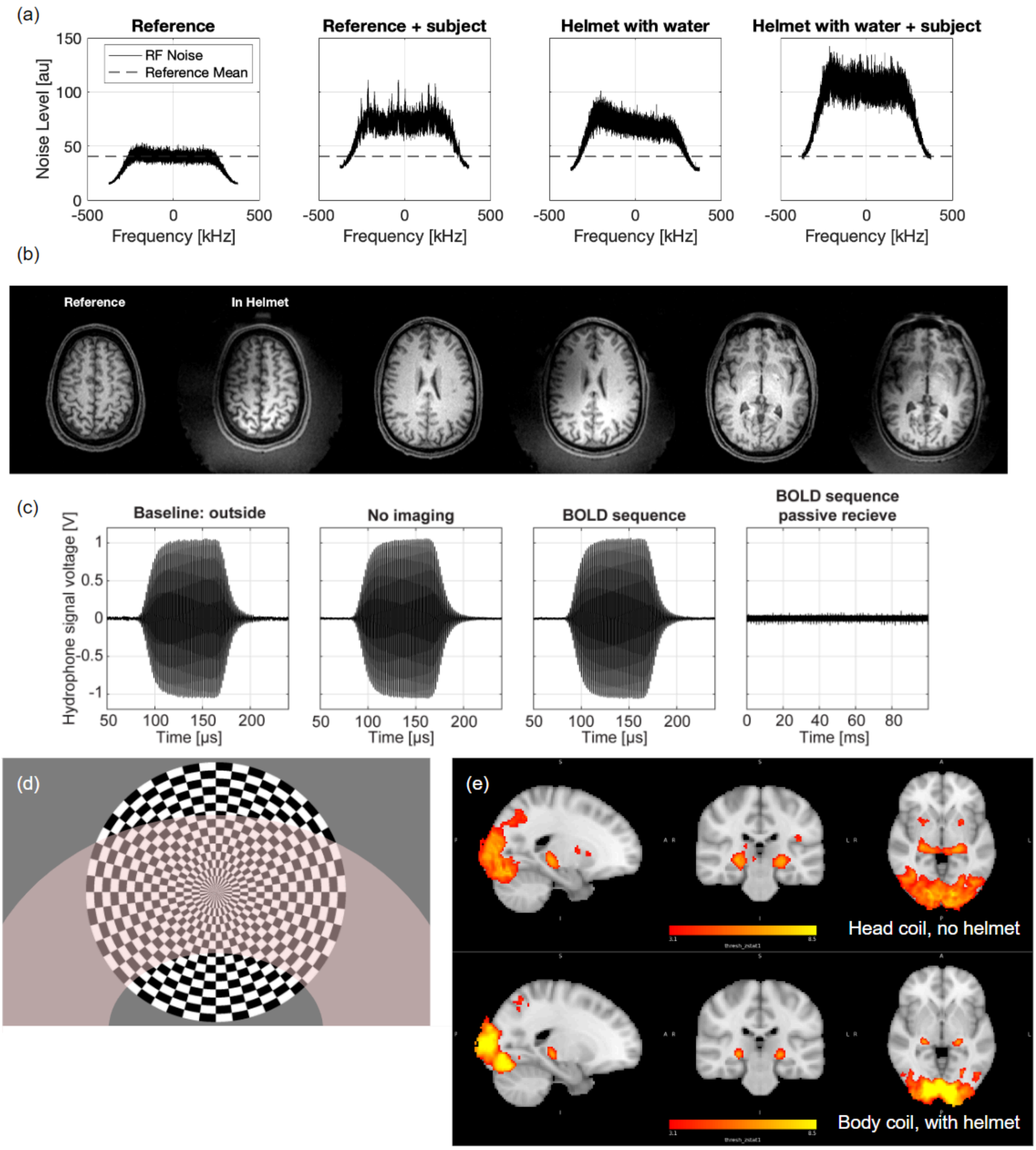

Figure S4: MR compatibility and imaging performance assessment
(a) Radio-frequency (RF) noise spectrum analysis of the MR system in various states with and without the helmet, and with and without the participant demonstrating good compatibility with the MR environment. **(b)** Pairs of body coil images (coronal slices) of the same participant with and without the water-filled helmet present. **(c)** Comparison of fibre-optic hydrophone measurements of the focal waveform acquired with 2 averages at 8V drive voltage, outside the MR bore, inside the MR bore in the absence of any imaging sequence, and inside the bore during the BOLD imaging sequence shows no change in acoustic waveform amplitude. Recording hydrophone voltage over a longer period (1 average) detected noise spikes which were small compared to the transmitted waveform amplitude. The noise equivalent voltage calculated as 3 standard deviations of the voltage in the absence of the acoustic signal was 15.5 ± 4 mV on average and did not significantly differ for the in bore recordings. **(d)** Radial checkerboard visual stimulus used to elicit functional activity in the LGN and visual cortex. The approximate field of view when the helmet is present is shown with the shaded area. **(e)** Mean activation during visual task using a standard 64-channel head and neck coil (top) and using the body coil with the helmet present (bottom) showing robust measurements despite the reduced field of view and sensitivity.

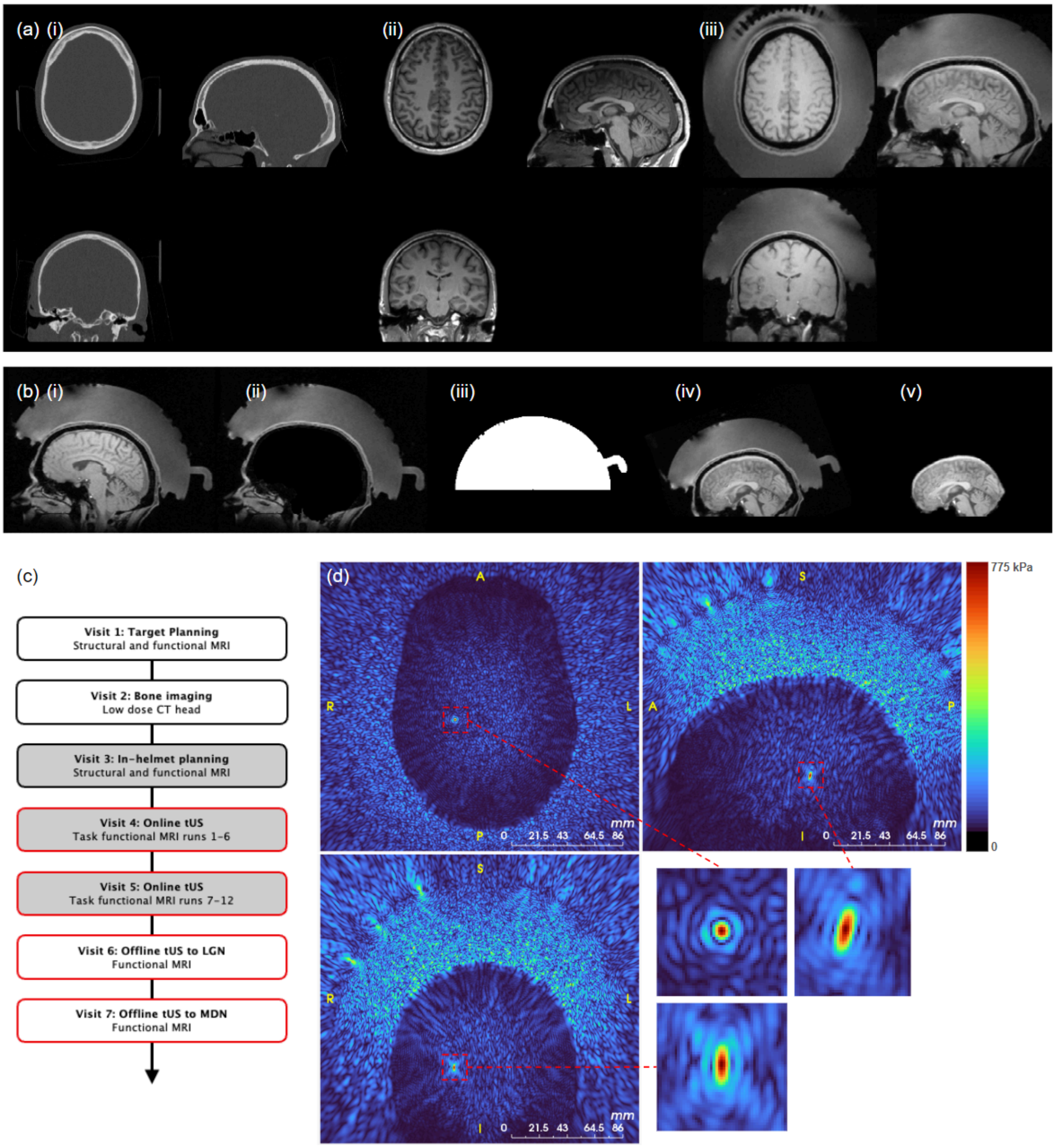

Figure S5: Registration workflow and experimental study design

(a) Example planning images after registration (i) CT, (ii) planning T1 image obtained in the head coil, and (iii) positioning T1 image obtained in the helmet with no magnetisation pulse. **(b)** The positioning T1 image (i) is brain extracted (ii) and then registered with a reference image of the helmet (iii) to move the image to helmet space (iv). In the stimulation sessions, the brain is then registered with the brain from the positioning session (v), and the registration used to calculate any shift in the position of the focus. **(c)** Study visits. **(d)** Simulated acoustic pressure field for a target in the right LGN for one participant.

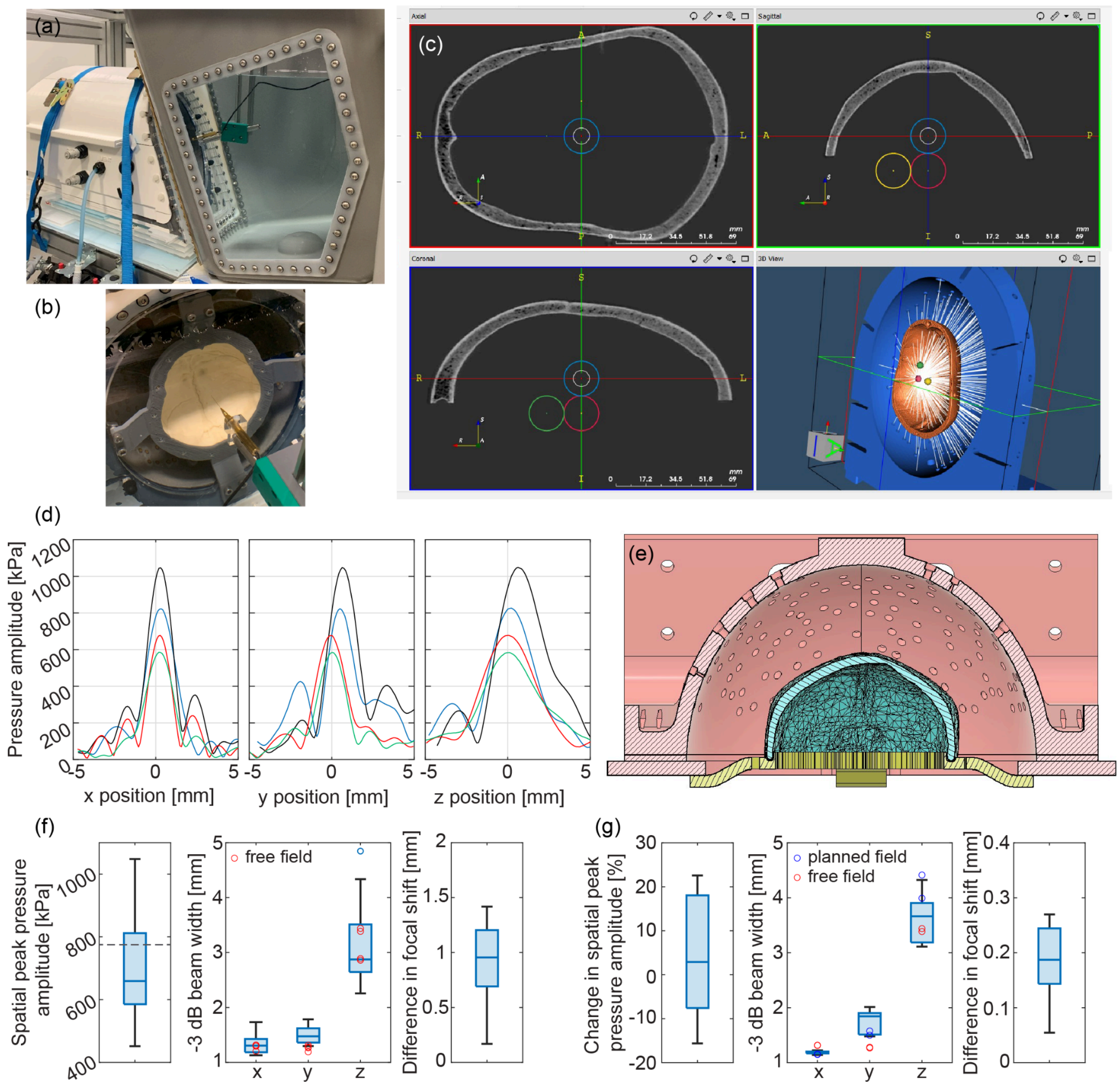

Figure S6: Ex vivo skull validation measurements and targeting accuracy
(a) Custom water tank field characterisation set-up. **(b)** Skull mounted in fixed position in array for skull validation measurements. **(c)** Screen-shot from k-Plan showing planned target positions inside an ex vivo human skull placed within the array. **(d)** Measured pressure amplitude profiles through the focus inside 4 skulls with planned position (0, 0, 0) and target pressure 750 kPa. **(e)** CAD drawing of the array housing with skull mount for registered geometry. The resulting stl file was used to register the position of the skull with the array elements for planning simulations. **(f)** Median and range (25th - 75th percentile) of spatial peak pressure amplitude, -3 dB beam width, and the position error (difference in focal shift) across 4 skulls with planned positions (0, 0, 0), (0, 0, 20), (0, 20, 0) and (20, 0, 0) mm. The intended target pressure was 750 kPa (black dotted line). The red dots show the focal dimensions in free field demonstrating focusing capability is not affected by the presence of the skull after aberration correction. Difference in focal shift is calculated from the absolute difference between the intended shift (20 mm) and the euclidean distance to the measured focal position. **(g)** Accuracy of replanning showing change in the spatial peak pressure amplitude, the -3 dB beam width, and the position error (difference in focal shift) when replanning was performed to geometrically shift the focal position by 5 mm from the planned focal position. Red dots show the focal dimensions in free-field.

Transducer and Drive System Parameters

	Manufacturer, Model Number	Centre Frequency	Ellipse Semi-axes	Aperture Diameter	Number of Elements	Element Distribution
Transducer	Custom array	555 kHz	x = 143 mm<br>y = 118.5 mm<br>z = 135 mm	x = 267 mm<br>y = 226 mm	256	3 mm planar elements pseudo-random on hemi-ellipsoid surface, water coupled
Matching	Custom electrical impedance matching network					
Drive system	Verasonics Vantage 256 + HIFU transmit config					

Driving System Settings

	Operating Frequency	Output level Setting	Focal Position Setting
Left LGN	555 kHz	23.0 ± 0.5 V	Subject specific model-based steering and aberration correction
MDN		18.4 ± 0.9 V	

Free Field Pressure Parameters

	Spatial Peak Pressure Amplitude	Position of Spatial Peak Pressure	Axial Focal Size (-3dB)	Lateral Focal Size (-3dB)	Axial Focal Size (-6dB)	Lateral Focal Size (-6dB)
Geometric centre	2.88 ± 0.06 MPa	[0, 0, 0] mm	3.4 mm	[1.3, 1.3] mm	4.9 mm	[1.8, 1.7] mm
Left LGN	2.58 ± 0.06 MPa	[-9, 23, -13] mm	1.8 mm	[1.4, 1.6] mm	2.6 mm	[2.0, 2.3] mm
MDN	2.25 ± 0.11 MPa	[2, 1, -5] mm	3.8 m	[1.2, 1.5] mm	5.1 mm	[1.7, 1.9] mm

Notes: Reference position is at centre (origin) of ellipsoid defining the array surface.

Pulse Timing Parameters

		Duration	Ramp Duration	Ramp Shape	Repetition Interval / Frequency
Experiment 1	Pulse	300 ms	10 ms	Cosine on voltage	3 s
	Pulse Train	15 s			24 s
	Pulse Train Repeat	8 min*			
Experiment 2	Pulse	20 ms	1 ms	Cosine on voltage	0.2 s / 5 Hz
	Pulse Train	80 s			

Notes: * 20 pulse train repeats: 10 active TUS, 10 sham in pseudo-random order

In Situ Exposure Parameters

	Spatial Peak Pressure Amplitude	Mean position of Spatial Peak Pressure	Axial Focal Size (-3dB)	Lateral Focal Size (-3dB)	Axial Focal Size (-6dB)	Lateral Focal Size (-6dB)
Left LGN	775 ± 163 kPa	[-9, 23, -13] mm	2.2 mm	[1.5, 1.5] mm	3.1 mm	[2.2, 2.2] mm
MDN	775 ± 163 kPa	[2, 1, -5] mm	3.0 mm	[1.5, 1.4] mm	4.1 mm	[2.0, 1.9] mm

Notes: Reference position is at centre (origin) of ellipsoid defining the array surface.

Mechanical Index	Maximum temperature rise in brain
1.04 ± 0.2	0.2 °C

Figure S7: Summary of acoustic parameters for the study

Output level setting and free field spatial peak pressure amplitude is given as mean ± standard deviation across participants for each target. The position of spatial peak pressure for the LGN and MDN is the mean position across participants. The in situ estimates of spatial peak pressure were obtained from treatment planning simulations run in k-Plan and are given as planned pressure ± relative error in measured spatial peak pressure compared to simulation obtained from the skull validation measurements. This error was also used to define the uncertainty in the reported MI. The maximum temperature rise in the brain was the maximum value obtained from thermal simulations across all participants. The difference in lateral focal size between x and y dimensions and the differences in axial focal sizes between target positions arises in large part because the focal ellipsoid is not aligned with the helmet axes for all locations.

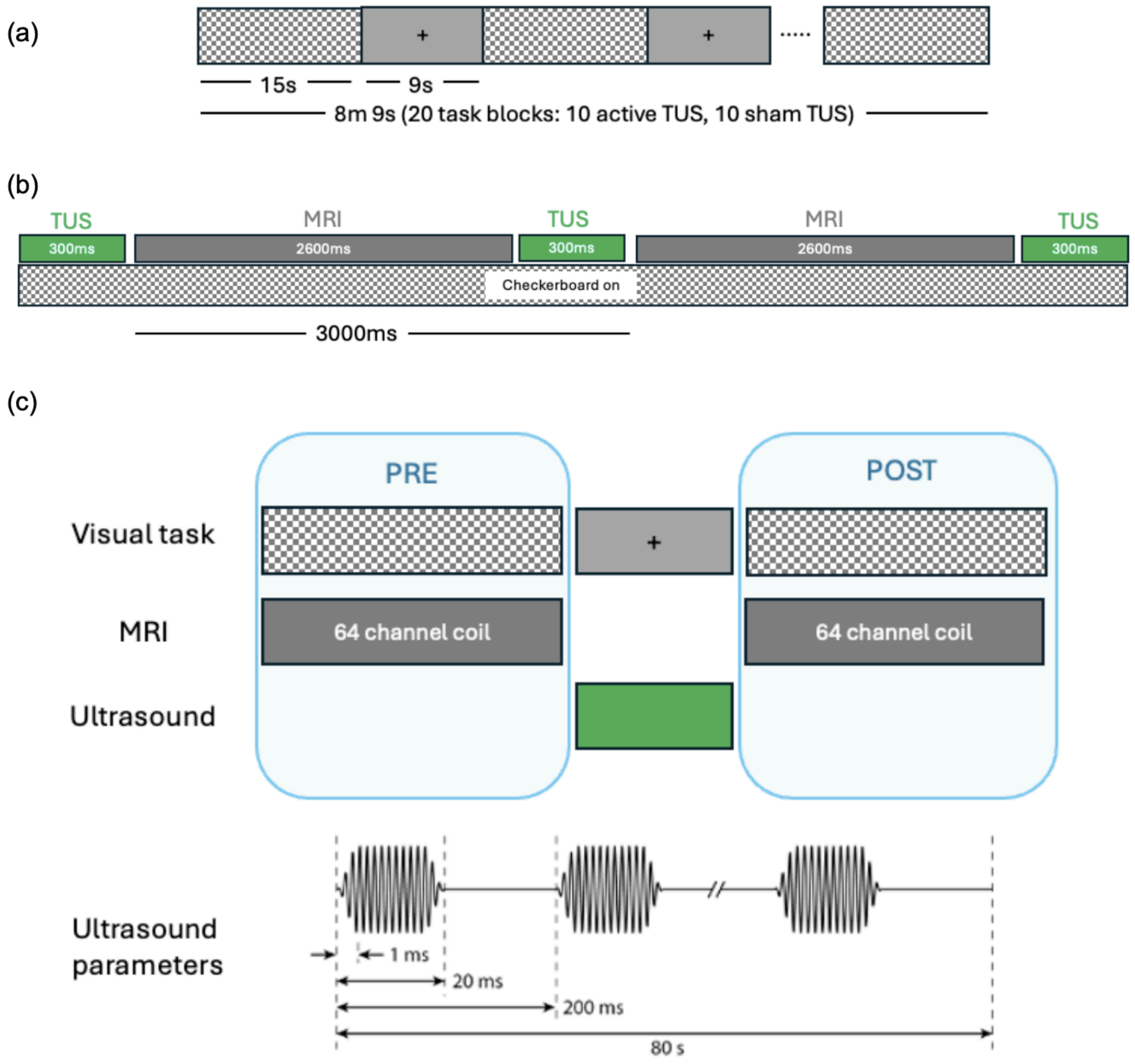

Figure S8: Experimental protocols for online and offline stimulation paradigms
(a) Block structure and duration for the online stimulation experiment. The checkerboard stimulus was presented in blocks of 15 seconds separated by 9 second blocks of rest. There were 20 checkerboard blocks in total, 10 with active TUS stimulation and 10 with sham stimulation. **(b)** During each active TUS checkerboard presentation block, the stimulation time (300ms) was followed by fMRI data acquisition (2.6s) at a repetition time (TR) of 3 seconds. **(c)** Timeline of offline stimulation experiment. fMRI data during checkerboard presentation (as described above) was collected before and after the offline TUS stimulation (duration 80 seconds with 20ms TUS pulses delivered every 200ms).

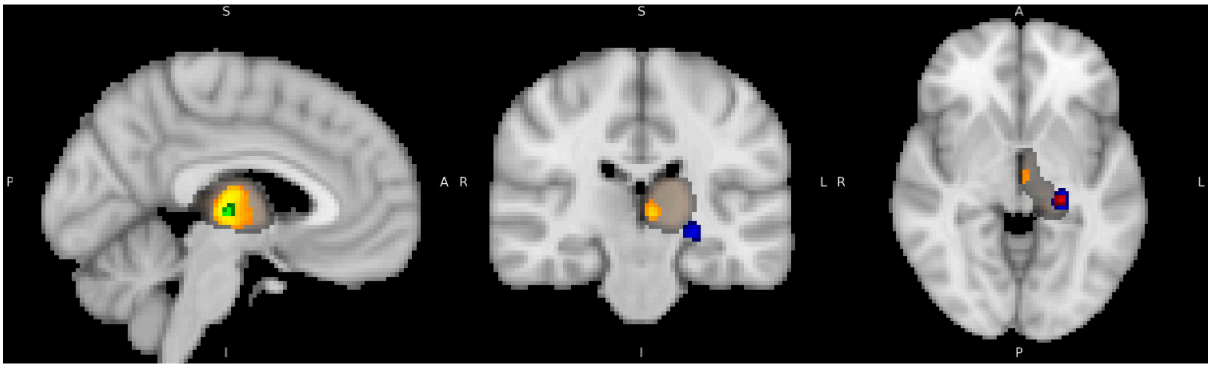

Figure S9: Anatomical targeting of thalamic nuclei across participants
Representation of the two stimulated thalamic nuclei. The two nuclei were determined through Freesurfer segmentation (LGN in blue, MDN in yellow). The stimulated area shown here was summed up for the four participants in the offline experiment (stimulated LGN in red, stimulated MDN in green). A thalamus map from the Harvard subcortical atlas is included in copper-grey.
